# Beyond the Walls: An Evaluation of a Pre-Release Planning (PReP) Programme for Sentenced Mentally Disordered Offenders

**DOI:** 10.3389/fpsyt.2018.00549

**Published:** 2018-11-02

**Authors:** Damian Smith, Susan Harnett, Aisling Flanagan, Sarah Hennessy, Pauline Gill, Niamh Quigley, Cornelia Carey, Michael McGhee, Aoife McManus, Mary Kennedy, Enda Kelly, Jean Carey, Ann Concannon, Harry G. Kennedy, Damian Mohan

**Affiliations:** ^1^National Forensic Mental Health Service, Central Mental Hospital, Dublin, Ireland; ^2^Department of Psychiatry, Trinity College, Dublin, Ireland; ^3^Irish Prison Service, Dublin, Ireland

**Keywords:** prison, mental health, homeless, continuity of care, transition, participatory action research

## Abstract

**Background:** Prison mental health services have tended to focus on improving the quality of care provided to mentally disordered offenders at the initial point of contact with the prison system and within the prison environment itself. When these individuals reach the end of their sentence and return to the community, there is an increased risk of morbidity, mortality, homelessness and re-imprisonment. New models of care have been developed to minimize these risks.

**Objectives:** The objective of this project was to establish a Pre-Release Planning (PReP) Programme with social work expertise, to enhance interagency collaboration and improve continuity of care for mentally disordered offenders upon their release. We aimed to evaluate the first 2 years of the programme by measuring its success at improving the level of mental health support and the security and quality of accommodation achieved by participants upon release in comparison to that reported at time of imprisonment. Additionally, we aimed to explore the impact of these outcomes on rates of re-imprisonment.

**Methods:** A process of participatory action research was used to develop and evaluate the first 2 years of the programme. This was a naturalistic prospective observational whole cohort study.

**Results:** The PReP Programme supported 43 mentally disordered offenders, representing 13.7%, (43/313) of all new assessments by the prison's inreach mental health service during the 2 years study period. When compared with that reported at time of reception at the prison, gains were achieved in level of mental health support (FET *p* < 0.001) and security and quality of accommodation (FET *p* < 0.001) upon release. Of those participants seen by the PReP Programme, 20 (46.5%, 20/43) were returned to prison during the 2-years study period. There was no significant relationship between re-imprisonment and gains made in mental health support (FET *p* = 0.23) or accommodation (FET *p* = 0.23).

**Conclusions:** We have shown that compared to that reported at time of reception at prison, the level of mental health support and the security of tenure and quality of accommodation both improved upon release following the intervention of the programme. Improved mental health support and accommodation were not associated with lower rates of re-imprisonment.

## Introduction

Prevalence rates for severe and enduring mental illnesses are significantly higher among sentenced prisoners than their peers in the general population (1–3). Mentally disordered offenders tend to have more complex health and social needs than non-mentally disordered offenders (4, 5).

Over the last decade, our service has developed a number of initiatives aimed at addressing the needs of mentally disordered offenders in remand (6, 7) and sentenced (8) prisons. These projects have been successful in improving the quality of care provided to these individuals at the initial point of contact with the prison system and within the prison environment itself.

The immediate post-release period however, is a time which poses increased risks for all prisoners, but especially those with a history of mental illness (9), including an increased risk of morbidity, mortality and homelessness (10–12). Moreover, in the context of the current homeless and housing crisis (13, 14) this vulnerable group are likely to be further marginalized and exposed to these adverse outcomes. Rates of re-imprisonment are high for all offenders both in Ireland (15) and worldwide (16). In relation to those offenders with a mental illness, rates of re-imprisonment are increased when compared with non-mentally disordered offenders (17).

When prisoners near the end of their sentence, a number of potential supports are available to them both internal and external to the prison. These are provided by the criminal justice and public health systems, as well as non-governmental organizations and the person's family network. These supports however, are typically fragmented and independent of one another, risking the individual falling through the gaps between services upon their release (18).

The World Health Organization has outlined a framework for patient-centered, integrated healthcare provision (19). This model emphasizes the need for collaboration between agencies and disciplines to improve patient outcomes and experiences, particularly for those with complex needs. These principles have been embedded in healthcare policy across the UK (20) and Ireland (21). Despite their complex healthcare needs, programmes for prison populations are conspicuous by their absence in these clinical strategies. It has been suggested that enhanced coordination between medical and mental health teams, and early identification of needs prior to release, can promote involvement of community based supports and assist in achieving continuity of care (22–24). These recommendations are echoed in Human Rights legislation. Of particular relevance is Rule 107 of the United Nations Standard Minimum Rules for the Treatment of Prisoners (The Nelson Mandela Rules), which highlights the importance of maintaining or establishing “*relations with persons or agencies outside the prison as may promote the prisoner's rehabilitation”* (25). However, efforts to establish and maintain relations with “*persons or agencies outside the prison”* can be challenging. The double stigma of being mentally ill and a convicted offender, along with high rates of substance misuse and homelessness (5, 26), can act as barriers to engagement with community based healthcare. It could also be argued that due to the complex social needs of mentally disordered offenders, that coordination of robust and holistic care plans should routinely be incorporated into prison inreach mental health services (27).

Various models have been proposed to overcome these challenges, most of which involve case management in the pre- and post- release periods for varying amounts of time (24). Assertive Community Treatment (ACT) has been utilized to provide intensive case management for up to 1 year in the post-release period (28). This intervention tends to be expensive and therefore more time limited approaches have been developed. Mckenna et al. have shown that a time limited intervention in the pre-release period based on the principles of ACT can improve engagement with community mental health services in the post-release period (29).

Critical Time Intervention (CTI) is a holistic approach to case management in the pre- and post-release period, which has demonstrated benefits in assisting mentally disordered offenders to engage with healthcare supports in the post-release period (22, 30, 31). CTI case managers aim to establish effective and trusting relationships with service users prior to their release from an institution in order to identify and ameliorate potential barriers to community reintegration (32, 33). Thereafter, they provide a time-limited period of support in the post-release period to help achieve transfer of care. In a randomized control trial of CTI within a prison setting, Jarrett et al. reported that the majority of the case manager's work in establishing support systems was performed within the prison, prior to the prisoner's release. Jarrett et al. also suggested that social workers may be best placed to fulfill the role of case manager due to the complex social problems faced by these individuals and the knowledge of local services and agencies needed to engage community supports (22).

The objective of this project was to establish a new Pre-Release Planning (PReP) programme involving case management by mental health social workers, to enhance interagency collaboration and improve continuity of care for sentenced mentally disordered offenders as they transition from prison to the community.

We aimed to evaluate the first 2 years of the PReP Programme by measuring its success at improving health and social outcomes for released mentally disordered offenders. In particular we aimed to explore for gains achieved in the level of mental health support and the security and quality of accommodation achieved by participants upon release in comparison to that reported at time of imprisonment. Finally, we aimed to explore the impact of these outcomes on rates of re-imprisonment.

## Methods

### Setting

This study took place in Ireland's oldest penal institution, Mountjoy Prison, which was opened in 1850. Mountjoy Prison is a closed, medium secure prison for adult males, and is the main committal prison for sentenced prisoners in Dublin city and county. It has capacity for 630 prisoners. The prison complex consists of the main prison, a training unit and a 10-bed High Support Unit (8).

### Study design

A process of participatory action research was chosen to design and develop the PReP Programme. Action research is described as a process involving a spiral of steps, each of which is composed of a cycle of planning, action and critical reflection (34). This process can result in organizational change and development. The authors have previously used this method to develop prison inreach mental health services (7, 8).

The initial “planning” step involved a literature review and was followed by an iterative process of identifying and consulting stakeholders then drafting and re-drafting the new model of care until there was sufficient support for the change process to proceed. Stakeholders included managers from the National Forensic Mental Health Service (a specialist tertiary mental health service funded and managed by the state health service), the Irish Prison Service, Probation Services, community based homeless support agencies, service users (prisoners availing of the support of the existing prison inreach mental health service) and their families. This series of stakeholder meetings and consultations led to the interactive development of a protocol for case finding and engagement, multi-agency liaison and interventions including the need for an integrated approach to release planning for mentally disordered offenders. Given the complex mental health and social needs of these individuals, social work expertise was identified as a vital, yet missing component of the exisiting inreach mental health service.

Subsequently, in March 2015, a social worker was redeployed from inpatient services at the National Forensic Mental Health Service, and the PReP Programme was established. A second social worker was added in November 2015 providing a 1.5 full time equivalent resource. Although case management was led by social workers, the PReP programme was supplemented by other members of the existing Mountjoy Prison Inreach Mental Health Service, which included two full time community forensic mental health nurses, a visiting consultant forensic psychiatrist, and 1–2 visiting psychiatric trainees.

This was a naturalistic prospective observational whole cohort study. The intervention of the programme was provided to all individuals on the inreach mental health service caseload within 12 months of their earliest date of release. Since its inception, the key interventions of the programme have evolved based upon feedback received from service users and family members at pre-release planning (PReP) meetings held prior to an individual's release. In addition stakeholders were afforded the opportunity to participate in critical reflection at weekly multiagency meetings.

### Interventions of the PReP programme:

**Figure 1:**
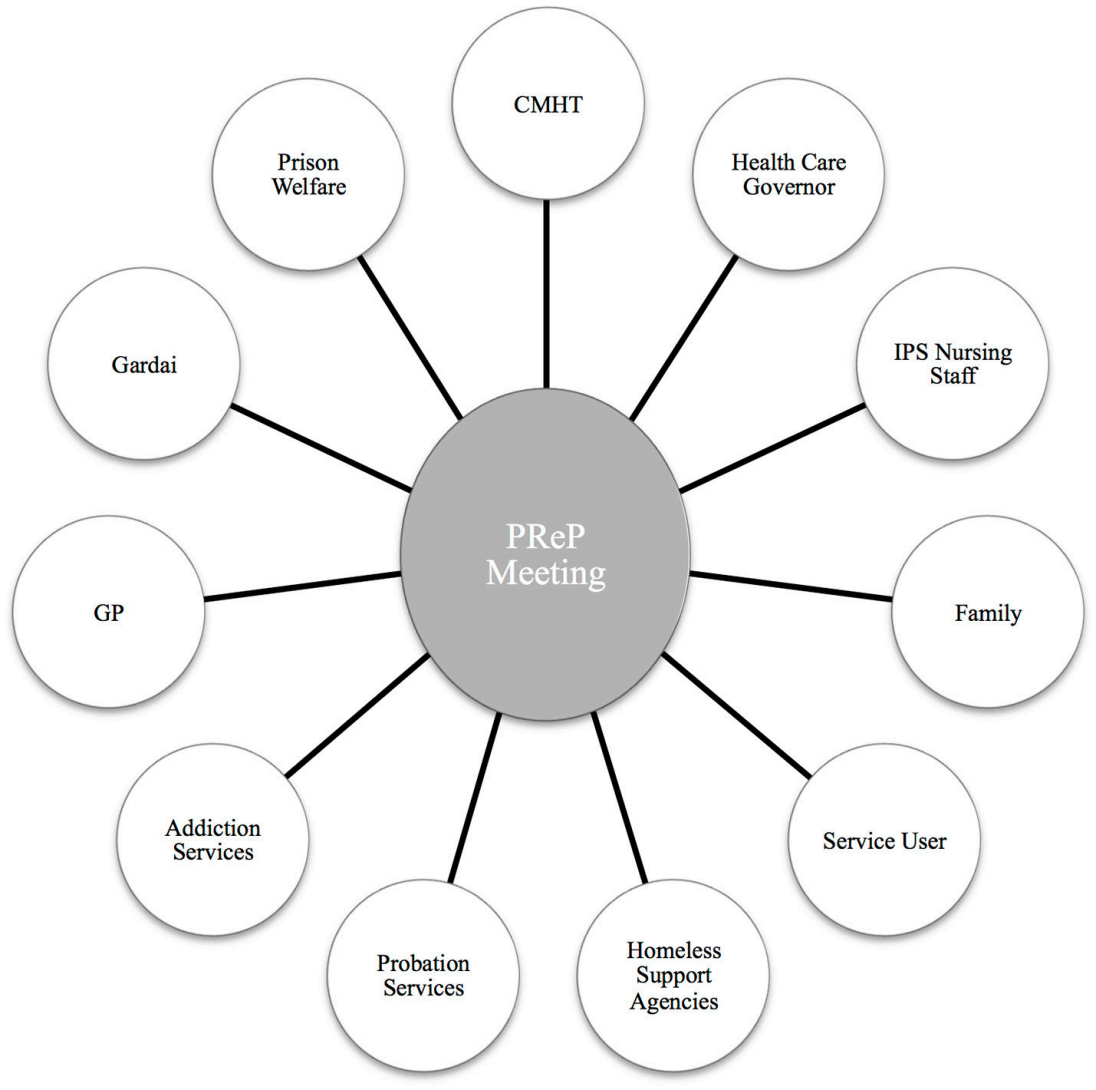
Examples of stakeholders invited to attend Pre-Release Planning (PReP) meetings prior to the individual's release. CMHT, Community Mental health Team; IPS, Irish Prison Service; GP, General Practitioner.

**Establishing trusting professional relationships with mentally disordered offenders in the pre-release period**.
**Liaison with mental health and other support agencies**—Establishing or maintaining relationships with community based mental health teams and other support agencies including: general practitioners, addiction services, intellectual disability services, accommodation providers, homeless support agencies and vocational programmes.
**Advocacy**—Addressing queries and concerns raised by community based mental health teams and other support agencies. In addition the programme advocated on behalf of participants to ensure social welfare payments and medical payment schemes were in place upon their release.
**Family support**—Providing information regarding diagnosis, treatment needs and relapse prevention. Exploring risks concerning the person on their return to the community including child protection issues and suitability of accommodation. This was of particular relevance for participants who planned to live with a family member on their release.
**Release planning**—Coordinating robust, holistic care plans prior to the person's release from custody. In most cases release plans were informed by multiagency, multidisciplinary pre-release planning (PReP) meetings held within 1 month of the person's release from prison. Figure 1 displays examples of the various stakeholders invited to attend PReP meetings. There was no statutory requirement for any stakeholder to attend pre-release meetings. Written release plans containing details of all relevant supports, contact details of key persons in the community, and accommodation arrangements were provided to all participants supported by the programme.
**6. Post-release support**—Providing time limited telephone support for service users, family members and receiving services, to ensure adequate handover and aid transition of care.
**7. Service evaluation through data collection and analysis**.

### Referral process and participants

During the study period, referrals to the Moutjoy Inreach Mental Health Service were received through multiple sources.

Upon reception at the prison, all newly received prisoners were screened by prison general nursing staff for a history of mental illness, active signs of mental illness and risk of harm to self or others. If a need for increased levels of observation was identified, the prisoner could be placed directly in the high support unit. In the event of a prisoner being placed in the high support unit, members of the Moutjoy Prison Inreach Mental Health Service aimed to assess them on the following working day.

All new committals were assessed by a prison general practitioner (GP) within 24 h of reception, and a referral generated to the Moutjoy Prison Inreach Mental Health Service if deemed necessary. Referrals were also received from other sentenced or remand prisons in the event of a prisoner with identified mental health needs being transferred to Moutjoy Prison.

Additionally, referrals of prisoners already allocated within the prison were received at weekly multiagency meetings chaired by the visiting consultant forensic psychiatrist and attended by the healthcare prison governor, the prison chief nurse officer, general prison nursing staff, probation services, prison psychology, prison general practitioner, and chaplaincy. Finally, family members and prisoners themselves also initiated referrals.

In the first instance all new referrals were assessed by the inreach mental health service's community forensic mental health nurses then triaged at weekly multi-agency meetings and appropriate follow up arranged.

As the PReP Programme social workers were an integral part of the Moutjoy Inreach Mental Health Service no formal referral was required. They engaged with any patient on the inreach team's caseload within 12 months of their earliest date of release. Participants on the programme were all those individuals on the Moutjoy Prison Inreach Mental Health Service caseload who were released to the community within the 2 years study period from 1st March 2015 to 28th February 2017.

### Variables, data sources and measurements

For all participants demographic and clinical information was routinely collected by members of the PReP Programme based on assessment and information gathered from electronic prison medical records and collateral sources. Binary measures were used when possible to aid with data analysis. Variables included age, nationality, offense type, homeless status, accommodation at time of reception to the prison, prior engagement with community mental health teams, lifetime history of self-harm, lifetime history of polysubstance abuse, lifetime history of psychosis, active psychosis at time of first assessment and ICD-10 (35) diagnosis at time of release. Diagnoses were documented by the Mountjoy Inreach Mental Health Service and PReP Programme based on serial clinical interviews and review of past medical and psychiatric case records from prison and community sources. All diagnoses were validated by a Consultant Forensic Psychiatrist.

Offense type related to the most serious index offense on reception at the prison and was classified as violent or non-violent. A violent offense was defined as an act of physical violence on a person and included homicide, assault, robbery, aggravated burglary, contact sexual offenses, false imprisonment, driving offenses involving injury to others and arson where there was a possibility of injury to others.

Homelessness was defined as rough sleeping or residence in homeless shelters reported at the time of committal. Rough sleeping was defined as sleeping outside on the street or in other open spaces. Those individuals staying with family or friends, or in long term placements were not included in the definition of homelessness for the purposes of this study. More detailed information about the security of tenure and quality of accommodation at time of reception and upon release was also captured.

Regarding outcome measures, the mental health/healthcare support and accommodation achieved on day of release was recorded. This information was gathered from interviews, collateral sources, electronic prison medical records and correspondence with receiving community based supports. In order to explore whether or not gains had been achieved following the intervention of the PReP Programme, in terms of level of mental health support and security of tenure and quality of accommodation, these outcomes were compared before and after the period of imprisonment. If a participant of the programme was re-imprisoned within the 2 years study period this was identified and recorded.

The DUNDRUM Toolkit (36) was used to assess the risk-appropriateness (whether transfer to a particular level of therapeutic security is necessary) of the mental health outcomes achieved upon release. DUNDRUM-1 (37) assesses level of security required. The DUNDRUM-2 (38) rates urgency of need for admission. The sum score of the DUNDRUM-1 is divided by the number of items to provide a mean score which is always between zero and four. A mean DUNDRUM-1 score >3 would guide a need for high therapeutic security, between 2 and 3 would guide toward medium therapeutic security and between 1 and 2 would guide toward acute low therapeutic security, often referred to as Psychiatric Intensive Care Unit. Scores lower than one indicate an open hospital ward or community setting would be appropriate. These scores are not binding but assist the clinical decision maker for individual cases. The mean scores for groups are useful guides to the appropriateness of patient placement from a risk-need appropriateness perspective to ensure proportionality and safety. The DUNDRUM-1 and DUNDRUM-2 have previously been used for this purpose in a remand prison setting (7).

DUNDRUM-1 and DUNDRUM-2 mean scores were calculated by members of the Moutjoy Prison Inreach Mental Health Service for all participants in the week prior to their release from custody.

### Ethical approval

The study protocol was approved by the National Forensic Mental Health Service Research, Audit, Ethics and Effectiveness Committee and by the Irish Prison Service Research Ethics Committee as a service evaluation project (39). In accordance with internationally recognized ethical principles, service evaluation studies do not require signed informed individual consent for all patients assessed and participating. Service evaluation is an ethical obligation in order to ensure appropriate use of resources, appropriate quality and standards for patients and continuous learning at the systems level. All patients therefore benefit. Nonetheless all participants gave written informed consent to participate in the programme. No randomization procedure was used for allocation to the PReP Programme. All data collected were anonymized and no individual patient data have been presented.

### Data analysis

Anonymized data were analyzed using IBM SPSS version 24. We used Chi-square tests to explore the relationship between categorical variables. A Fisher Exact test was used when there was an expected count of <5 in any of the groups. We used *t*-tests to compare continuous variable means between two groups and a one-way analysis of variance (ANOVA) when comparing means between multiple groups.

## Results

**Figure 2:**
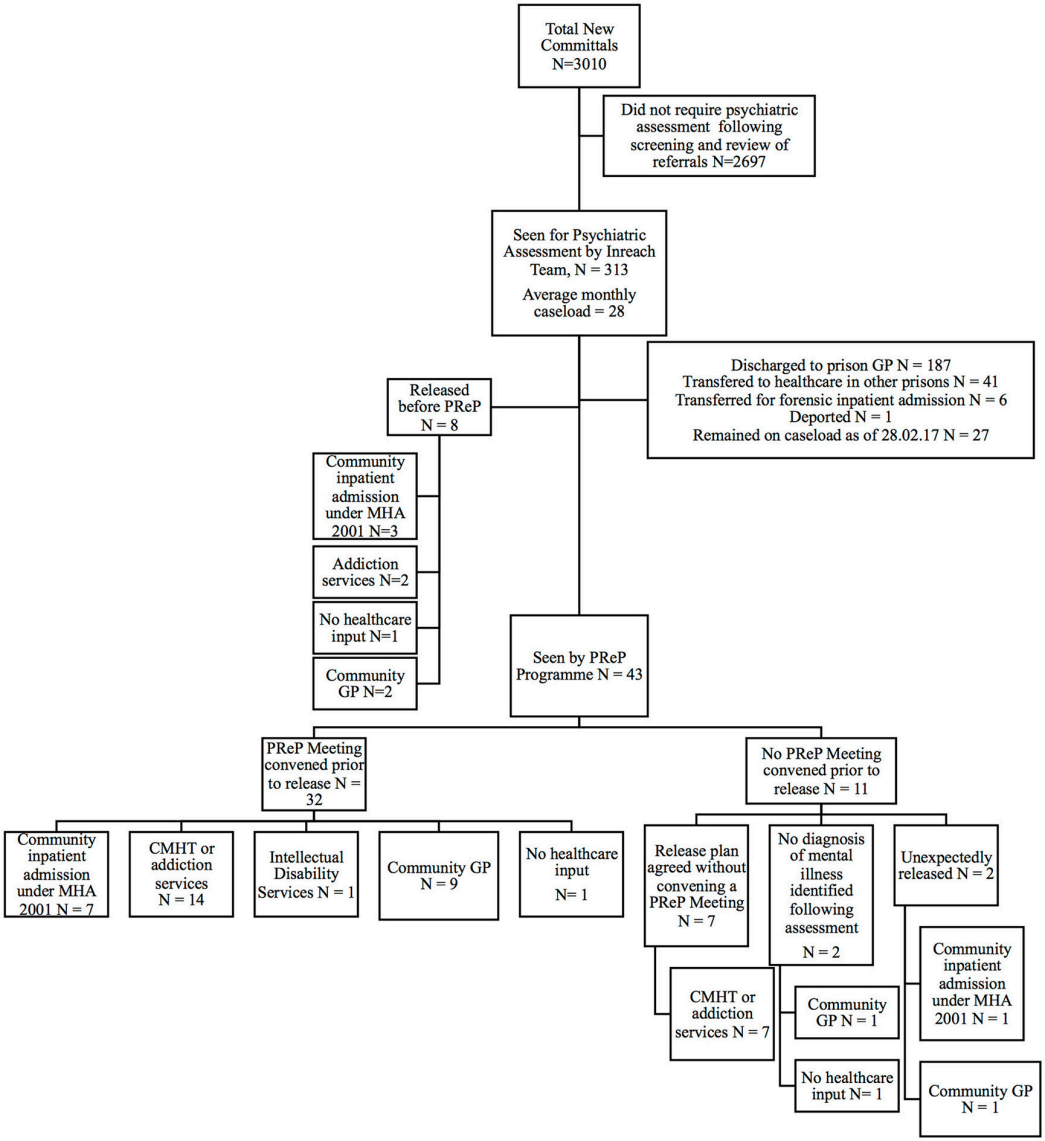
Consort diagram displaying mental health outcomes on day of release for all those seen by the Mountjoy Prison Ireach Mental Health Service and PReP Programme from 1st March 2015 to 28th February 2017. PReP, Pre-Release Planning; CMHT, Community Mental Health Service; GP, general practitioner; MHA 2001, Mental Health Act 2001.

Figure 2 displays the pathway from point of reception at the prison to mental health outcome on day of release for all 3,010 committals to Mountjoy Prison, from 1st March 2015 to 28th February 2017. Of these, 2,697 committals (89.6%, 2697/3010) were deemed not to require psychiatric assessment following screening of referrals by the Mountjoy Prison Inreach Mental Health Service. The remaining 313 (10.4%, 313/3010) committals were taken onto the caseload; 43 (13.7%, 43/313) of whom were subsequently supported by the PReP Programme as they were expected to be released within 12 months. This represented 40 individuals as one participant was imprisoned at Mountjoy Prison twice and another three times, during the study period.

For this group, the median duration from date of initial reception at any prison during the relevant committal episode to date of release was 516 days (*N* = 43, mean 672.9 days SD 772.0), and from date of committal to Mountjoy Prison to date of release was 259 days (*N* = 43, mean 534.6 days SD 722.7). The median duration from date of committal at Mountjoy Prison to date of first assessment by the Inreach Mental Health Service was 6 days (*N* = 43, mean 54.8 days SD 164.7). The median duration from date of committal at Mountjoy Prison to date of first assessment by the PReP Programme was 124.0 days (*N* = 43, mean 380.1 days SD 696.3). The median duration from date first seen by the PReP Programme to date of release was 123 days (*N* = 43, mean 154.4 days SD 149.2).

Mental health outcomes for the eight individuals on the caseload who were released before being seen by the PReP programme are also displayed in Figure 2. For this group the median duration from date of committal to Mountjoy Prison to date of first assessment by the Mountjoy Prison Inreach Mental Health Service was 2.5 days (*N* = 8, mean 3.9 days SD 4.5). These individuals had a median duration in Mountjoy Prison of 5.5 days (mean 15.9 days SD 18.1). Despite spending only a brief period in Mountjoy Prison the majority (87.5%, 7/8) of this group were referred for healthcare follow up upon release by the inreach mental health service.

A pre-release planning (PReP) meeting was convened prior to release for 32 of those availing of the support of the programme (74.4%, 32/43). Ten (31.3%, 10/32) of these meetings were attended by community mental health teams, 17 (53.1%, 17/32) were attended by a family member/spouse, and nine (28.1%, 9/32) were attended by the patient themselves.

A meeting was not convened for the remaining 11 committals for the following reasons: a release plan had already been agreed by all parties (*N* = 7); the patient was unexpectedly released (*N* = 2); no severe mental illness (defined as major depressive disorder, hypomania, bipolar disorder and/or any form of psychosis including schizophrenia, schizoaffective disorder and any other non-affective, non-organic psychosis) was identified following serial assessments by the team (*N* = 2). The mental health outcomes for these 11 patients are shown in Figure 2. Ten of these individuals had healthcare support arranged on the day of their release despite no formal meeting having been held. No healthcare input was arranged for the remaining individual as they were found not to meet criteria for a severe mental illness.

All 43 committals seen by the PreP Programme were issued with a written release plan, the contents of which are described in the methods section of this article. In the event of healthcare follow up being arranged a written release plan was also forwarded to the receiving healthcare provider.

### Case description

Demographic, legal and clinical characteristics for those who availed of the support of the PReP Programme (*N* = 43) and those who did not (*N* = 8) are displayed in Table 1. Participants and non-participants did no differ significantly in relation to age, nationality, homeless status at time of reception or clinical variables. Participants however, were more likely to have been charged with a violent offense, to have been transferred from another prison and to have had a previous admission to a secure forensic psychiatric hospital.

**Table 1:** Comparison of demographic, legal and clinical characteristics of participants and non-participants of the PReP Programme.

	**Participants (*****N*** = **43)**		**Non-Participants (*****N*** = **8)**		**Statistical test of difference**	***p*-value**
	***N* (%)**	**Mean (SD)**	***N* (%)**	**Mean (SD)**	
Age		35.67 (8.02)		32.88 (7.75)	*t* = 0.91	0.37
**NATIONALITY**						
Irish	41 (95)		6 (75)		FET	0.11
Non-Irish	2 (5)		2 (25)		
**HOMELESS ON RECEPTION**						
Yes	21 (49)		2 (25)		FET	0.27
No	22 (51)		6 (75)		
**OFFENSE TYPE**						
Violent	22 (51)		0 (100)		FET	0.02
Non-violent	21 (49)		8 (0)		
**TRANSFERRED FROM ANOTHER PRISON**						
Yes	31 (72)		0 (0)		FET	< 0.001
No	12 (28)		8 (100)		
**PREVIOUS ADMISSION TO SECURE HOSPITAL**						
Yes	18 (42)		0 (0)		FET	0.04
No	25 (58)		8 (100)		
**PSYCHOTIC AT FIRST ASSESSMENT**						
Yes	16 (37)		5 (62)		FET	0.25
No	27 (63)		3 (38)		
**LIFETIME PSYCHOSIS**						
Yes	33 (77)		6 (75)		FET	1.00
No	10 (23)		2 (25)		
**HISTORY OF PSA**						
Yes	39 (91)		7 (88)		FET	1.00
No	4 (9)		1 (12)		
**HISTORY OF SELF-HARM**						
Yes	26 (60)		4 (50)		FET	0.70
No	17 (40)		4 (50)		
**PREVIOUS CONTACT WITH CMHT**						
Yes	30 (70)		7 (88)			0.42
No	13 (30)		1 (12)		FET

*PSA, polysubstance abuse; CMHT, Community Mental Health Team; FET, Fisher's exact test*.

### Demographics

Of the 43 committals seen by the PReP Programme, all were male, and 41 (95.3%, 41/43) identified themselves as Irish, with the remaining two individuals identifying as Non-Irish Europeans. The mean age at time of first assessment by the Mountjoy Prison Inreach Mental Health Service was 36 years (SD 8.0, range 21–63).

### Offense type

Regarding the nature of the most serious index offense, of those seen by the PReP Programme 48.8% (21/43) were charged with a violent offense, that is one involving physical violence to another person. The remaining 51.2% (22/43) were charged with non-violent offenses. Thirty-one (31/43, 72.1%) of those supported by the PReP Programme were transferred from another remand or sentenced prison to Mountjoy Prison. Two were re-patriated from prisons abroad.

### Contact with children and child protection issues

Sixteen of the 43 committals seen by the PReP Programme reported having children. Of these, 14.0% (6/43) reported that they had contact with their children prior to reception at prison. As a result of concerns regarding risk posed to children in the event of release, a total of seven referrals were made to Tusla, Ireland's Child and Family Agency, by members of the PReP Programme in keeping with their obligations under Ireland's child protection legislation.

## Clinical characteristics

### Primary ICD-10 diagnoses, active and lifetime psychosis

Table 2 displays the primary ICD-10 diagnosis at the time of release for all those seen by the PReP Programme. Almost two thirds of those seen had primary ICD-10 diagnoses of Schizophrenia, Schizotypal and Delusional Disorders (58.1%, 25/43) or Bipolar Affective Disorder (2.3%, 1/43). An additional 16.3% (7/43) had a primary diagnosis of a drug induced psychotic episode. At the time of initial assessment by the Mountjoy Prison Inreach Mental Health Service, 37.2% (16/43) of those seen following screening and referral were assessed as being actively psychotic. Based on information from interview and collateral sources, just over three quarters of individuals seen by the PReP Programme had a lifetime history of a psychotic illness (76.7%, 33/43).

**Table 2:** Primary ICD-10 diagnosis at time of release for all those seen by the PReP Programme (*N* = 43).

	**Primary ICD-10 diagnosis**	***N***	**%**
F00-09	Organic disorders	1	2.3
	- Alcohol related dementia	
F10-19	Substance use disorder
	- Drug induced psychosis	7	16.3
	- Polysubstance abuse only	1	2.3
F20-29	Schizophreniform disorders
	- Schizophrenia	18	41.9
	- Schizoaffective disorder	5	11.6
	- Delusional disorder	2	4.7
F30-39	Mood disorder
	- Manic episode	1	2.3
	- Depressive episode	3	7.0
F60-69	Personality disorder
	- Emotionally unstable personality disorder	3	7.0
F70-79	Mild intellectual disability	2	4.7
	Total	43	100

*ICD-10, International Statistical Classification of Diseases and Related Health Problems, 10th Revision*.

### Co-morbidity and self-harm history

Almost all individuals supported by the programme had a lifetime history of polysubstance abuse (90.7%, 39/43). Based upon collateral information, one quarter (25.6%, 11/43) had a co-morbid diagnosis of a personality disorder. Of all those seen, 60.5% (26/43) had a lifetime history of deliberate self-harm.

### Previous contact and engagement with community mental health teams and other healthcare supports

The majority of those seen by the PReP Programme (69.8%, 30/43) reported prior contact with a community mental health team at some point before their reception at the prison. Eighteen individuals (41.9%, 18/43) had previously been admitted to the Central Mental Hospital, the Republic of Ireland's only secure forensic hospital.

Regarding level of engagement with mental health supports prior to reception, 14 (32.6%, 14/43) had no contact with any mental health supports; six (14.0%, 6/43) were attending a general practitioner alone; 20 (47%, 20/43) were attending outpatient services (community mental health team, addiction services or intellectual disability services), one was in hospital (2.3%, 1/43) and two had been repatriated from international prisons (4.7%, 2/43).

Regarding compliance with prescribed psychiatric medications, of the 25 (58.1%, 25/43) committals prescribed such treatment prior to their imprisonment, 14 reported being fully compliant (56.0%, 14/25), seven (28.0%, 7/25) reported being partially compliant and four reported being non-compliant (16.0%, 4/25).

### Outcomes following the intervention of the PReP programme:

1. **Mental health outcomes:**
  Mental health supports arranged on day of release for all those seen by the PReP Programme are displayed in Figure 2.
  Of the 43 committals seen by the programme, 35 (81.4%, 35/43) were referred for community mental health team follow up upon release, of which 82.9% (29/35) were accepted. Fifteen (51.7%%, 15/29) of these accepted referrals, were initially declined. In these cases further efforts were made by the PReP Programme to liaise with the receiving service to address their concerns so that the referral process could be completed.
  Table 3 displays a comparison between the level of healthcare support at time of reception at prison compared with that arranged on day of release following the intervention of the PReP Programme. A Fisher Exact Test indicated that the level of mental health support significantly improved upon release from prison, following the intervention of the programme (FET *p* < 0.001).
  Regarding post-release engagement with arranged mental health supports, the PReP Programme confirmed that 89.7% of those accepted by community mental health teams (26/29) attended their first appointment in the post-release period. Of these, 27.6% (8/29) were admitted involuntarily to a general psychiatric hospital under the Mental Health Act 2001.
  Receiving mental health services were then contacted in the post-release period to confirm if the referred individual remained engaged following attendance at their first appointment. The median duration of post-release follow up was 20.5 days (mean 61.31 days, SD 104.09). At time of follow up, 20 individuals (76.9%, 20/26) remained engaged with community mental health teams, of whom four were inpatients, and none had returned to prison.
  **Risk-appropriateness of arranged mental health supports:**
  Mean DUNDRUM-1 triage security and DUNDRUM-2 triage urgency scores for those seen by the PReP Programme (*N* = 43) released to community inpatient (*N* = 8), outpatient services (community mental health team, addiction services, intellectual disability services) (*N* = 22), general practitioner (*N* = 11) and no healthcare follow up (*N* = 2) are summarized in Table 4.
  Mean DUNDRUM-1 triage security scores (ANOVA *F* = 1.99, between groups df = 3, within groups df = 39, *p* = 0.13) and DUNDRUM-2 triage urgency scores (ANOVA *F* = 1.87, between groups df = 3, within groups df = 39, *p* = 0.15), although not significant, tended to be higher for those transferred to higher levels of mental health support.
2. **Accommodation outcomes:**
  Twenty one (48.8%, 21/43) committals seen by the PReP Programme were homeless at the time of their reception to prison. This included five (23.8%, 5/21) who reported rough sleeping, 13 (61.9%, 13/21) who reported staying in emergency “night to night” homeless shelters and two (9.5%, 2/21) who reported staying in short term, “week to week” homeless shelters. The remaining individual (4.8%, 1/21) was an inpatient in a general psychiatric hospital prior to reception at prison, but had no regular accommodation before this and reported staying in emergency homeless shelters. Twenty-one participants (48.8%, 21/43) continued to meet the definition of homelessness at the time of release. No individuals were released to rough sleeping.
  Table 5 displays a comparison between accommodation at time of reception at prison compared with that achieved on day of release following the intervention of the PReP Programme. A Fisher Exact Test indicated that the security of tenure and quality of accommodation significantly improved upon release from prison following the intervention of the PReP Programme (FET *p* < 0.001).
3. **Re-imprisonment:**
  Of those participants seen by the PReP Programme, 20 (46.5%, 20/43) were returned to prison during the 2-years study period. The median duration from date of release to end of the study period was 274.0 days (mean 314.0 days SD 185.9 days). Fifteen individuals (34.9%, 15/43) were under the supervision of probation services when initially released, 7 (46.7%, 7/15) of who were re-imprisoned during the 2-years study period.
  Table 6 displays rates of re-imprisonment for all those supported by the PReP Programme according to the level of mental health support and accommodation achieved on day of release. There was no significant relationship between re-imprisonment and gains made in level of mental health support (FET *p* = 0.23) or accommodation (FET *p* = 0.23) following the support of the PReP Programme, however the duration of follow up was relatively short (median 274.0 days).

**Table 3:** Comparison of level of healthcare support at time of reception to prison with that on day of release, following the intervention of the PReP Programme (*N* = 43).

	**Healthcare support**					**Total**
	**None**	**GP**	**Outpatient services (CMHT, Addiction services, ID services)**	**Prison**	**Hospital**
Prior to reception at prison (*N*)	14	6	20	2	1	43
On day of release (*N*)	2	11	22	0	8	43

*CMHT, Community Mental Health Team; ID, intellectual disability; GP, general practitioner*.

**Table 4:** Risk-appropriateness of mental health outcomes for all those seen by PReP Programme (*N* = 43).

	***N* (%)**	**D-1 triage security score**		**D-2 triage urgency score**	
		**Mean (SD)**	**95% CI**	**Mean (SD)**	**95% CI**
Psychiatric admission	8 (19)	2.11 (0.60)	1.61–2.62	2.05 (0.71)	1.46–2.64
Outpatient Services (CMHT, Addiction services, ID services)	22 (51)	1.64 (0.84)	1.27–2.01	1.45 (1.00)	1.01–1.89
GP	11 (25)	1.54 (0.80)	1.00–2.07	1.32 (1.16)	0.54–2.10
No healthcare follow-up	2 (5)	0.70 (0.57)	−4.38–5.78	0.35 (0.21)	−1.56–2.26

*D-1, DUNDRUM-1; D-2, DUNDRUM-2; SD, standard deviation; 95% CI, 95% confidence interval; CMHT, Community Mental Health Team; ID, intellectual disability; GP, general practitioner*.

**Table 5:** Comparison of accommodation at time of reception to prison with that on day of release, following the intervention of the PReP Programme (*N* = 43).

	**Accommodation**					**Total**
	**Rough sleeping**	**Emergency/Short term hostel**	**Long term hostel, secure tenancy, living with family**	**Hospital**	**Prison**
Prior to reception at prison (*N*)	5	15	20	1	2	43
On day of release (*N*)	0	16	19	8	0	43

*Emergency Hostel Accommodation, in a homeless shelter booked on a nightly basis; Short Term Hostel, accommodation in a homeless shelter booked on a weekly basis; Long Term Hostel, accommodation in a homeless shelter booked for 6 months or longer; Secure Tenancy, private rented accommodation or own home*.

**Table 6:** Impact of level of mental health support and accommodation outcomes on rates of re-imprisonment, following the intervention of the PReP Programme (*N* = 43).

**Re-imprisoned?**	**Healthcare support on day of release**				**Total**
	**None**	**GP**	**Outpatient Services (CMHT, Addiction services, ID Services)**	**Involuntary Hospital admission under MHA 2001**
Yes (*N*)	2	4	12	2	20
No (*N*)	0	7	10	6	23
	**Accommodation on day of release**			
	**Rough sleeping**	**Emergency/short term hostel**	**Long term hostel, secure tenancy, living with family**	**Involuntary hospital admission under MHA 2001**
Yes (*N*)	0	10	8	2	20
No (*N*)	0	6	11	6	23

*MHA 2001, Mental Health Act 2001; CMHT, Community Mental Health Team; ID, intellectual disability; GP, general practitioner*.

### Secondary analysis

For the reasons outlined above, eleven participants availed of the support of the PReP Programme but did not have a pre-release planning (PReP) meeting prior to their release. A secondary analysis was performed to explore if a meeting was associated with any difference in outcome measures. There was no significant difference found between those who had a meeting (*N* = 32) and those who did not (*N* = 11) in relation to mental health outcomes (FET *p* = 0.24), security of tenure and quality of accommodation achieved upon release (FET *p* = 0.74) and rates of re-imprisonment (*X*^2^ = 0.38, df = 2, *p* = 0.72).

## Discussion

We have followed a participatory action research design to introduce a new service for mentally disordered offenders as they transition from prison to the community. We have completed an evaluation of the first 2 years of the project to examine whether the goals of the service were achieved. In particular whether those referred to the PReP Programme had improved levels of mental health support and improved security of tenure and quality of accommodation upon their release in comparison to that reported at time of imprisonment. During the period of this study, there were no other major changes in the organization, management or delivery of prison in-reach services nor was there any major change in the organization, management or delivery of prison and criminal justice services.

### Summary of findings

We have shown that compared to that reported at time of imprisonment, the level of mental health support and the security of tenure and quality of accommodation both improved following the intervention of the PReP Programme. In the absence of a control group we cannot show that the PReP programme caused this effect, but we believe this is so. Higher levels of mental health support and improved accommodation were not associated with lower rates of re-imprisonment within the 2 years study period however the follow up period was relatively short. We were not able to further analyse relationships between variables and outcomes owing to lack of statistical power.

### Strengths and limitations

This project, the first of its kind in Ireland, embodies the principles of integrated and multidisciplinary healthcare provision. Post-release mental health and accommodation outcomes were mapped for all those seen by the PReP programme. Healthcare outcomes were also mapped and presented for eight patients on the inreach mental health team's caseload who were released prior to availing of the support of the PReP programme.

Prior to the development of the PReP Programme, release planning in the prison studied was performed by a medically focused inreach mental health service comprised of doctors and nurses. The addition of mental health social work expertise enhanced the ability of the team to develop robust release plans in collaboration with community based supports. As suggested by Jarrett et al. (22) social workers might be best placed to coordinate such care plans given their knowledge of local services and support agencies. The social workers of the Pre-Release Planning (PReP) Programme were based within the prison as part of the inreach mental health team. This allowed them to build trusting relationships with mentally disordered offenders in the pre-release period. Practical supports offered by the programme, including liaison with family members and assistance in accessing accommodation and social welfare may have acted as incentives for engagement before and after release. This may have been reflected by the high rates of engagement with arranged mental health appointments immediately after release (89.7%, 26/29).

The main focus of the programme was to improve pre-release planning and manage transfer of care to community based supports. Social workers from the programme subsequently offered time limited telephone support to service users, family members and receiving services. This correspondence revealed that the majority of those receiving mental health follow up from community mental health services remained engaged at a median duration of 3 weeks following their release (76.9%, 20/26). Unlike Assertive Community Treatment (ACT) and Critical Time Intervention (CTI), the programme did not provide case management in the post-release period. Although this may be viewed as a limitation of the PReP Programme, previous studies (22, 29, 40) and a recent systematic review (24), have highlighted the importance of pre-release planning in any intervention to aid the transition for mentally disordered offenders. We acknowledge that the less intense follow up provided by our programme results in difficulty determining the quality of engagement with mental health and other supports in the post-release period. Future projects will focus on assessing whether or not the achievements of the PReP programme translate into long term sustained improvements in engagement with mental health supports, accommodation and legal outcomes.

Homelessness is one of the greatest challenges facing released prisoners (23) and may act as an impediment to engaging with healthcare supports (41). These individuals may be further marginalized losing out on available accommodation to family's and non-mentally ill persons experiencing homelessness. Although rates of broadly defined homelessness were not reduced following the intervention of the programme (*N* = 21 on reception vs. *N* = 21 on day of release), there was evidence of improvements in the security of tenure and quality of accommodation obtained upon release. Moreover, the fact that more individuals were not released to homelessness may represent an improved outcome, given that previous studies have highlighted an increased risk of homelessness and unstable housing upon release from prison (42). Despite improvements in both the level of healthcare support and accommodation achieved following imprisonment and the intervention of the PReP Programme, 46.5% (20/43) of those supported by the intervention were re-imprisoned within the 2 years study period. Although disappointing, this rate of re-imprisonment is consistent with that reported for general prison populations in our jurisdiction (15). Gains made in healthcare and accommodation outcomes were not associated with reduced rates of re-imprisonment during a relatively short follow up period. This finding may not be surprising as a number of more intensive post-release case management models have found an association with increased rates of re-imprisonment through the increased level of monitoring provided by these interventions in the post-release period (24). Regrettably, information was not available regarding the status of participant's mental illness and level of engagement with community mental health supports at the time of re-imprisonment.

A process of participatory action research was used to design, develop and evaluate the PReP programme. This design meant that the programme could be implemented without delay following the identification of a need by stakeholders within the prison. Although this creates practical advantages for service development, it may result in difficulty identifying the specific variables asscoiated with achieved outcomes.

At the planning stage of the project, a multidisciplinary, multiagency pre-release planning (PReP) meeting was envisaged to be a central component of the intervention provided by the PReP Programme. Despite this not all of those supported by the programme had a pre-release planning meeting. We have outlined reasons why meetings were not convened for eleven of the forty-three participants. We also performed a secondary analysis to explore if a meeting was associated with improved outcomes and found that it was not. We believe this is an interesting observation. It implies that the networking and liaison work carried out by PReP team members is as effective as a meeting arranged in addition to that liaison work, at least from a quantitative, outcomes point of view. It remains possible that better qualitative outcomes and experiences would result from the addition of a meeting as outlined in previous studies of this kind (40). This may be a focus of future research by our service.

In the event of a pre-release planning meeting being held, attendance by community mental health teams, families and service users was relatively poor. In Ireland, as in many developed countries, there is no statutory requirement for any agency to attend pre-release planning meetings. Unfortunately community mental health teams were often unable to attend due to scheduling problems and on occasion due to reluctance to accept the individual until late in the prisoner's sentence. Despite having the support of prison authorities it often proved difficult to transfer prisoners from their location in the prison to the site of pre-release planning meetings. This occurred mainly due to prison officer shortages or because the prisoner was too unwell to attend.

All mentally disordered offenders on the inreach mental health services' caseload released within the period studied were eligible for support by the programme. This inclusive approach did not permit the creation of a comparable control group, which would have allowed for more rigorous analysis regarding the effectiveness of the intervention. Additionally, the service has been operational for 2 years, therefore we were not able to further analyse relationships between variables and outcomes owing to a lack of statistical power. Also, the inclusive and real-world nature of this project resulted in some participants availing of the support of the programme despite not meeting criteria for a mental illness at the time of release.

This project was set in an all male sentenced prison and its findings may not be transferable to female prison populations. Future plans by our service include the establishment of a similar social work-led PReP Programmes in a number of Ireland's other sentenced prisons, including its main female prison.

## Conclusions

We have shown that compared to that reported at time of imprisonment, the level of mental health support and the security of tenure and quality of accommodation both improved at time of release, following the intervention of the PReP Programme. Higher levels of mental health support and improved accommodation were not associated with lower rates of re-imprisonment within the 2 years study period.

## Data availability

The raw data supporting the conclusions of this manuscript will be made available by the authors, without undue reservation, to any qualified researcher.

## Author contributions

DS completed the first and revised drafts of the manuscript, which were then edited by SHa, AF, SHe, NQ, CC, DM and HK who also assisted with data analysis. The intervention was designed and developed by DM, SHa, AF, SHe and PG, with assistance from all stakeholders including prisoners and their families. All authors contributed to the participatory action research process. All authors read and approved the final manuscript.

### Conflict of interest statement

The authors declare that the research was conducted in the absence of any commercial or financial relationships that could be construed as a potential conflict of interest.

## Abbreviations

PReP: pre-release planning
CMHT: community mental health team
GP: general practitioner
CTI: critical time intervention
ACT: assertive community treatment.
